# Unveiling the Cardioprotective Potential of Hydroxytyrosol: Insights from an Acute Myocardial Infarction Model

**DOI:** 10.3390/antiox14070803

**Published:** 2025-06-28

**Authors:** Alejandra Bermúdez-Oria, Eugenia Godoy, Virginia Pérez, Camila Musci Ferrari, Martin Donato, Juan Fernández-Bolaños, Tamara Zaobornyj, Verónica D’Annunzio

**Affiliations:** 1 Department of Food Phytochemistry, Instituto de la Grasa (Spanish National Research Council, CSIC), Ctra. de Utrera km. 1, Pablo de Olavide University Campus, Building 46, 41013 Seville, Spain; j.fb.g@csic.es; 2 Facultad de Farmacia y Bioquímica, Instituto de Bioquimica y Medicina Prof. Alberto Boveris (IBIMOL, UBA-CONICET), Universidad de Buenos Aires, Buenos Aires 1113, Argentina; mvperez@fmed.uba.ar (V.P.); camilamuscif@gmail.com (C.M.F.); mdonato@fmed.uba.ar (M.D.); tamaraz@ffyb.uba.ar (T.Z.); 3 Facultad de Medicina, Departamento de Patología—Instituto de Fisiopatología Cardiovascular, Universidad de Buenos Aires, Buenos Aires 1113, Argentina; egn.godoy@gmail.com

**Keywords:** antioxidant, by-products, cardioprotection, hydroxytyrosol (HT), ischemic heart disease (IHD), mitochondrial function

## Abstract

Cardiovascular diseases remain the leading cause of death worldwide, highlighting the urgent need for novel therapeutic strategies. The Mediterranean diet is renowned for its cardiovascular benefits, largely attributed to extra virgin olive oil (EVOO) and its phenolic compounds, particularly hydroxytyrosol (HT). HT, a potent antioxidant and anti-inflammatory agent, has demonstrated significant therapeutic potential in mitigating myocardial damage following acute myocardial infarction (AMI). However, there is a notable lack of published evidence regarding the effects of HT administration in the context of acute ischemia/reperfusion (I/R) injury, making this study a novel contribution to the field. This study aimed to evaluate the cardioprotective effects of HT using the Langendorff technique in an isolated mouse heart ischemia/reperfusion (I/R) model. Mice were administered a single intraperitoneal dose of HT (10 mg/kg) 24 h prior to the I/R protocols, and parameters such as the infarct size, mitochondrial function, and redox balance were assessed. The results revealed a remarkable 57% reduction in infarct size in HT-treated mice compared to untreated controls. HT treatment also improved mitochondrial bioenergetics, as evidenced by the increased membrane potential (ΔΨm), enhanced oxygen consumption, and reduced hydrogen peroxide (H_2_O_2_) production. Furthermore, HT restored the activity of the mitochondrial respiratory complexes, notably Complex I, even under I/R conditions. These findings highlight the efficacy of HT in reducing oxidative stress and preserving mitochondrial function, critical factors in cardiac disease. In conclusion, HT emerges as a promising therapeutic agent for ischemic heart disease, demonstrating both preventive and restorative potential. Future research should explore its clinical applicability to advance cardiovascular disease management.

## 1. Introduction

The Mediterranean diet is increasingly recognized for its health benefits, particularly its protective effects against cardiovascular disease, inflammation, and cancer [1]. One of the main components of this diet that has been shown to be responsible for those beneficial effects is the extra virgin olive oil (EVOO). In fact, the advantageous effects associated with the frequent intake of EVOO, such as the reduced risk of cardiovascular disease, are widely described and acknowledged [2].

Nowadays, cardiovascular diseases, and specifically ischemic heart disease, are the most frequent pathologies with the highest morbidity and mortality rates [3]. Early reperfusion therapy is crucial in clinical practice to prevent irreversible myocardial injury by restoring normal oxygen levels in tissues [4]. However, this maneuver is responsible for additional myocardial damage, namely, reperfusion injury involving the formation of reactive oxygen species. In reperfusion, mitochondrial damage is one of the determinants of the loss of cell viability and contractile function of cardiomyocytes. Several signaling pathways are involved in cardioprotective strategies tested to date [5]. This is why the use of myocardial cardioprotective strategies against ischemia/reperfusion injury (I/R) has gained increasing interest in the last decades; however, many of these fail when translated into the clinical setting [6]. These pathways converge in mitochondria and aim to preserve their function after I/R and to provide energy to sustain ventricular function after the infarction [7,8].

A healthy diet is one of the factors evaluated as protective of the myocardium against acute myocardial infarction. EVOO is olive juice whose composition is 98% saponifiable fraction (predominantly monounsaturated FAs, especially oleic acid), and the rest is represented by the unsaponifiable fraction (hydrocarbons, aliphatic and aromatic alcohols, phenols, sterols, tocopherols, liposoluble vitamins, volatile organic compounds, aldehydes, triterpenic acids, etc.). Although these latter molecules occupy a small percentage of EVOO, they account for the nutritional and biological properties [9]. Noteworthy, olives have a high content of phenolic compounds that are not completely transferred to the EVOO. Most of these molecules are concentrated in the remains after milling to produce olive oil due to their polarity. That is why the phenolic fraction of olive oil is less than 2% of the total phenolic content of the fruit, and the remaining 98% is retained in the by-products [10]. The olive oil industry represents one of the most important productive sectors worldwide, with Spain being the largest producer of olive oil in the world. In fact, Spain alone generates more than 5 million tons of olive oil extraction waste per season. In this context, it is important to emphasize the high added value of this industry’s products. Specifically, numerous studies focus on the recovery of phenolic compounds from by-products, since these molecules are known for their biological properties and their potential for the prevention of diseases [11]. As examples, oleuropein, oleocanthal, hydroxytyrosol (HT), and tyrosol have shown biological activities as antioxidant, anti-inflammatory, or anticancer agents [12,13,14].

HT is a simple phenol, and it is the main olive oil antioxidant with a potent anti-inflammatory activity [15,16]. The European Food Safety Authority (EFSA) allows the health claim that ≥5 mg HT/day prevents low-density lipoprotein (LDL) oxidation and therefore reduces the risk of atherosclerosis [17]. Studies have demonstrated its ability to attenuate oxidative stress, reduce inflammation, and improve endothelial function, all of which are central to the pathogenesis of ischemic heart disease [18,19,20,21,22]. Moreover, HT exhibits multifaceted mechanisms of action, including the modulation of cellular signaling pathways implicated in liver or myocardial I/R injury [23,24,25]. However, it has not yet been studied whether the acute administration of HT can exert a protective effect against an I/R episode and, if so, the underlying mechanisms involved.

This study aims to investigate the cardioprotective effects of HT in a murine model of acute myocardial infarction (AMI) induced by I/R. Specifically, it seeks to evaluate the impact of a single intraperitoneal administration of HT (10 mg/kg) 24 h before euthanasia to produce I/R in isolated mouse hearts using the Langendorff technique. This study explores the influence of HT on infarct size, mitochondrial redox balance, mitochondrial bioenergetics, and the activity of mitochondrial respiratory complexes in the context of I/R injury. Ultimately, the findings will contribute to the understanding of the therapeutic potential of HT in reducing ischemic heart disease.

## 2. Materials and Methods

### 2.1. Materials

HT was extracted and purified from olive by-products using a chromatographic system following the processes described by Fernández-Bolaños et al. (2013) [26].

### 2.2. Animal Care

All procedures performed in these studies involving animals were in accordance with the ethical standards of the Animal Care and Research Committee of the University of Buenos Aires (CICUAL UBA RESCD-2023-1423-UBA-DCT#FMED). C57BL male mice were housed in ventilated cages with a 12 h light/dark cycle and controlled temperature (20–22 °C) and were fed with normal chow and water ad libitum.

### 2.3. Treatment of Mice with HT

Mice were administered a single intraperitoneal injection of 10 mg/kg HT 24 h prior to euthanasia. The dose of 10 mg/kg was chosen based on previous studies, such as Pei et al. [25], which demonstrated the efficacy of a 20 mg/kg dose for reducing infarct size in a rat model of ischemia/reperfusion injury. Considering the dose range used in their study and the fact that their protocol involved administering HT 5 min prior to ischemia, we selected a lower dose of 10 mg/kg to balance efficacy with a more feasible treatment schedule. The 24 h pre-treatment period allowed sufficient time for HT to exert its systemic antioxidant and mitochondrial protective effects. The mice were divided into 4 groups for the experiments.

Control group: Untreated mice.
I/R group: Mice subjected to I/R.
HT group: Mice treated with HT at the specified dose.
HT + I/R group: Mice treated with HT at the specified dose and subjected to I/R.

### 2.4. Isolated Mouse Hearts

**Figure 1:**
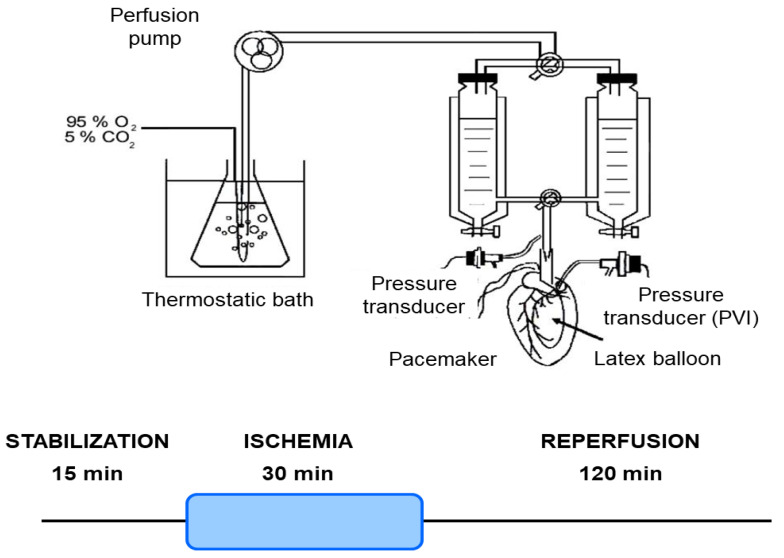
Schematic representation of the isolated heart Langendorff perfusion system and ischemia/reperfusion protocol. This diagram illustrates the experimental setup for maintaining isolated mouse hearts under controlled perfusion conditions. Key components include a thermostatic bath for temperature regulation, a perfusion pump for precise buffer delivery, and a pacemaker/pressure transducer to monitor cardiac function. The system ensures constant retrograde perfusion of the coronary arteries with oxygenated Krebs–Henseleit buffer. Hearts underwent a 15 min stabilization period, followed by 30 min of global ischemia, and then 120 min of reperfusion.

Mice were euthanized, and their hearts were isolated and perfused using the Langendorff technique, as previously outlined [27]. Briefly, mice were anesthetized with an intraperitoneal injection of sodium pentobarbital (150 mg/kg) and sodium heparin (500 IU/kg). The heart was then perfused with Krebs NaHCO_3_-buffered solution containing 118.5 mM NaCl, 4.7 mM KCl, 24.8 mM NaHCO_3_, 1.2 mM KH_2_PO_4_, 1.2 mM MgSO_4_, 1.5 mM CaCl_2_, and 10 mM glucose, bubbled with 95% O_2_ and 5% CO_2_ (pH 7.4) at 37 °C. A pacemaker (QRS Ingeniería Médica, La Plata, Argentina) was connected to the heart to stabilize the heart rate at 4620 ± 17 beats/min. The coronary perfusion pressure (CPP) was monitored using a pressure transducer (Argon Medical Devices, Plano, TX, USA) connected to the perfusion line. The heart was perfused at a constant flow rate of 4.1 ± 0.1 mL/min (Figure 1).

### 2.5. Ischemia/Reperfusion Protocol

The myocardial infarction protocol consisted of 30 min of global no-flow ischemia after 15 min of stabilization, followed by 120 min of reperfusion for infarct size analysis. For the heart mitochondrial function analysis, the protocol consisted of 30 min of global no-flow ischemia after 15 min of stabilization, followed by 60 min of reperfusion (Figure 1).

### 2.6. Infarct Size Measurement

The assessment of infarct size was conducted using 2,3,5-triphenyltetrazolium chloride (TTC), as previously described [28]. After 120 min of reperfusion, the hearts were frozen and sliced into 1 mm transverse sections from apex to base. These sections were then incubated for 20 min in 1% TTC solution (pH 7.4, 37 °C) and subsequently fixed in 10% formalin. Viable sections were stained red, while non-stained sections indicated the infarcted area. The sections were traced onto acetate sheets and analyzed using planimetry (Image Pro Plus, version 4.5). Infarct size was expressed as a percentage of the left ventricular area [26].

### 2.7. Heart Mitochondrial Function

#### 2.7.1. Heart Mitochondria Isolation and Mitochondrial Membrane Preparation

Heart mitochondria were isolated from mouse heart homogenates through a series of steps involving differential centrifugation using a refrigerated centrifuge (Sorvall-Instruments-Du Pont, Wilmington, CA, USA). Initially, the left ventricles were minced in ice-cold Tris–HCl-EGTA (STE) buffer containing 250 mM sucrose, 10 mM Tris–HCl, and 2 mM EGTA at pH 7.4. A brief digestion was carried out in STE buffer supplemented with 0.5% (*w*/*v*) fatty acid-free bovine serum albumin (BSA), 5 mM MgCl_2_, 1 mM ATP, and 2.5 U/mL type XXIV bacterial proteinase. Following a 4 min digestion at 4 °C, 5 volumes of STE buffer was added, and each heart was homogenized using a Potter–Elvehjem glass-Teflon homogenizer (Sigma-Aldrich, San Luis, MO, USA) before being centrifuged at 8000× *g* for 10 min. The resulting pellet was resuspended in ice-cold STE buffer and centrifuged at 700× *g* for 10 min. Subsequently, the pellet was discarded, and the mitochondria were precipitated through two 10 min centrifugation rounds at 8000× *g*. Finally, the mitochondria were suspended in STE buffer at a concentration of approximately 10 mg of protein/mL. The protein concentration was determined with the Bradford reagent using bovine serum albumin as the standard.

Mitochondrial membranes were obtained by 3 cycles of freezing and thawing of the mitochondrial preparation and homogenized by passage through a 25-gauge hypodermic needle [29].

#### 2.7.2. Mitochondrial Oxygen Consumption

Oxygen uptake was assessed using polarography with a Clark-type electrode in a 1 mL chamber at 30 °C. Mitochondria were incubated in an air-saturated respiration medium composed of 120 mM KCl, 5 mM KH_2_PO_4_, 1 mM EGTA, 3 mM HEPES, and 1 mg/mL BSA, supplemented with 2 mM malate and 5 mM glutamate and adjusted to pH 7.2. Oxygen consumption was measured during both state 4 (respiration at rest) and state 3 upon the addition of ADP (active respiration) [30]. The respiratory control ratio (RC) was calculated as the ratio of state 3 to state 4 respiratory rates. Oxygen uptake was expressed in ng-atom O/min x mg protein.

#### 2.7.3. Hydrogen Peroxide Production

Hydrogen peroxide (H_2_O_2_) production was evaluated fluorometrically (365–450 nm) using the Amplex red fluorescent probe at 30 °C in a plate reader (Varioskan LUX, Thermo Scientific, Waltham, MA, USA) [31]. The reaction mixture comprised 125 mM sucrose, 65 mM KCl, 10 mM HEPES at pH 7.2, 2 mM KH_2_PO_4_, 0.01% BSA, 5 mM glutamate/malate, 0.5 U/mL HRP (horseradish peroxidase), 25 μM Amplex Red, and heart mitochondria (0.02–0.05 mg protein/mL). A calibration curve was generated using H_2_O_2_ (0.05–0.35 M). H_2_O_2_ production was expressed as nmol H_2_O_2_/min mg protein.

#### 2.7.4. Mitochondrial Inner Membrane Potential

The electrical component of the electrochemical potential (Δμ_H+_), namely, the mitochondrial membrane potential (ΔΨ), was analyzed by measuring the fluorescence of Rhodamine 123 (Rh-123) at 503–527 nm (λexc–λem) at 37 °C using a plate reader (Varioskan LUX, Thermo Scientific). A calibration curve was established using Rh-123 (0.01–0.015 μM) as the standard.

Initially, the fluorescence of the reaction medium containing 150 mM sucrose, 5 mM KH_2_PO_4_/K_2_HPO_4_, 20 mM K-HEPES (pH 7.2), and 0.1 μM Rh-123 was determined to calculate the total probe concentration ([Rh-123]_total_, in nmol/μL). Subsequently, freshly isolated mitochondria (0.1 to 0.3 mg protein/mL) were introduced into the media supplemented with 2 mM malate and 5 mM glutamate, in the absence of ADP, to establish metabolic state 4. In another set of experiments, mitochondria were added to the media in the presence of 0.5 mM ADP, aiming to establish metabolic state 3. After 1 min of incubation to reach equilibrium, the mitochondrial suspension was centrifuged at 15,000× *g* to pellet the mitochondrial fraction. The Rh-123 concentration remaining in the media ([Rh-123]_out_, in nmol/μL) was calculated from the fluorescence values of the supernatant. The concentration of probe incorporated into mitochondria ([Rh-123]_mit_, in nmol/μL) was calculated by the difference between the initial total amount of Rh-123 ([Rh-123]_total_) and the amount remaining in the media ([Rh-123]_out_). The concentration of free Rh-123 in the mitochondrial matrix ([Rh-123]in, in nmol/μL) was determined using the equation and the binding partition coefficients at 37 °C (K_i_ = 26 μL/mg, K_o_ = 120 μL/mg) [32]:

[Rh-123]_mit_ = K_i_ [Rh-123]_in_ + K_o_ [Rh-123]_out_

Finally, the mitochondrial membrane potential was calculated using the electrochemical Nernst–Guggenheim equation:

ΔΨ = 59 log ([Rh-123]_in_/[Rh-123]_out_)

#### 2.7.5. Mitochondrial Electron Transfer Activities

The enzyme activity of Complex I was determined at 550 nm using a plate reader (Varioskan LUX, Thermo Scientific) with an extinction coefficient (ε) of 19 mM/cm at 30 °C, with mitochondrial membranes suspended in 100 mM KH_2_PO_4_/K_2_HPO_4_ buffer at pH 7.4. Complex I activity, also known as NADH-cytochrome c reductase, was determined. Mitochondrial membranes were incubated with 0.2 mM NADH along with 25 μM cytochrome c^3+^ and 0.5 mM KCN. The enzymatic activities were expressed as nmol of reduced cytochrome c/min mg of protein [33]

### 2.8. Statistical Analysis

The results are expressed as mean values ± standard error of the mean (SEM). STATGRAPHICS^®^ Plus software version 19 (Statgraphics Technologies, Inc., The Plains, VA, USA) was used for statistical analysis. Comparisons among samples were made using one-way analysis of variance (ANOVA) and the Least Significant Difference (LSD) method, and a *p*-value < 0.05 was considered significant. For pairwise comparisons between two groups as shown in Figure 1, an independent Student’s *t*-test was applied.

## 3. Results

### 3.1. Infarct Size

**Figure 2:**
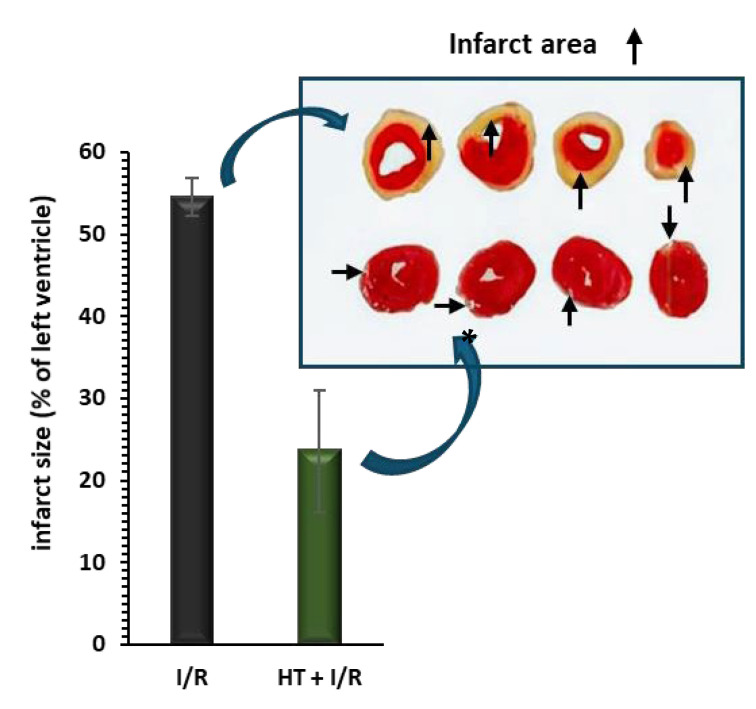
Infarct size, measured as a percentage of the total left ventricular area, was assessed following ischemia/reperfusion (I/R) injury using the triphenyltetrazolium technique. Pre-treatment with HT (10 mg/kg) 24 h prior to I/R resulted in a significant reduction in infarct size compared to the I/R group. For the I/R protocols, ischemia was induced for 30 min followed by 120 min of reperfusion. Data are presented as mean ± standard error of the mean (SEM). Statistical significance is indicated as follows: *p* * < 0.001 vs. I/R (n _I/R group_ = 7; n _HT + I/R group_ = 4). An independent Student’s *t*-test was used for comparisons between groups. Representative images of TTC-stained heart sections. Infarct area (pale) is clearly distinguishable from viable myocardium (red).

Figure 2 shows the behavior of the infarct size. Compared to the I/R protocol group, the administration of HT 24 h prior to euthanasia results in a significant reduction in the area of infarction induced by the I/R protocol (23.6 ± 7.4% vs. 54.5 ± 2.3%, *p* < 0.001).

### 3.2. Heart Mitochondrial Function

#### 3.2.1. Mitochondrial Oxygen Consumption

**Figure 3:**
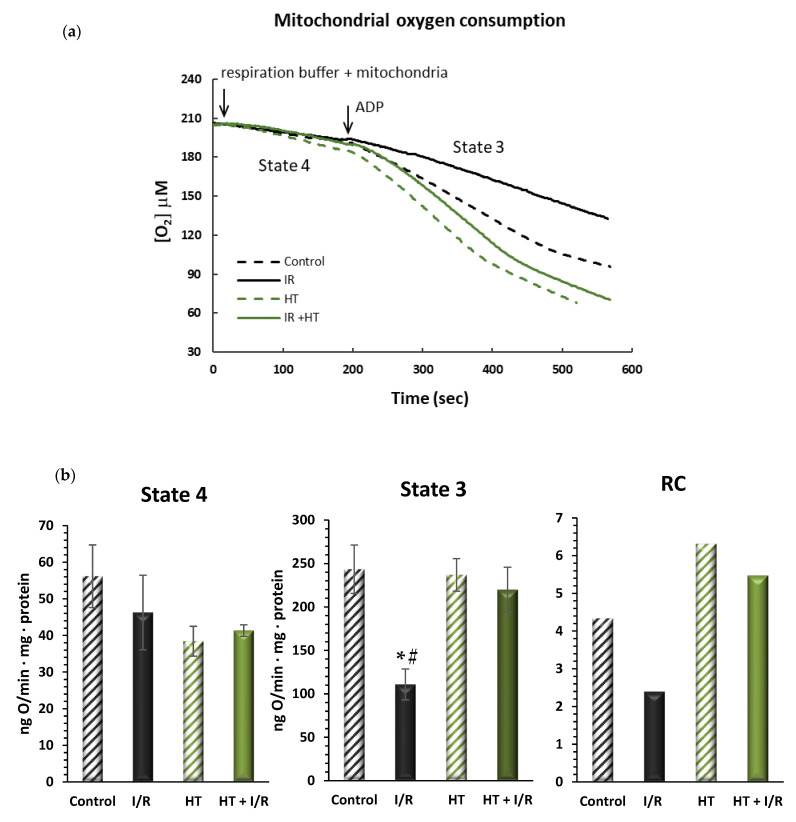
(**a**) Representative traces obtained from Clark-type oxygen electrode measurements illustrating μM oxygen consumption over time for the four experimental groups: control, I/R, HT, and HT + I/R. Each trace displays state 4 respiration (basal oxygen consumption) and the subsequent increase in oxygen uptake upon ADP addition, leading to state 3 respiration (ADP-stimulated respiration). (**b**) Mitochondrial function of the left ventricles was evaluated in control (untreated mice), I/R (mice subjected to 30 min of ischemia and 60 min of reperfusion, I/R), HT (mice treated with HT at the specified dose 24 h previously with lack of I/R protocol), and HT + I/R (mice treated with HT at the specified dose 24 h previously and subjected to I/R). The state 4 and state 3 mitochondrial oxygen consumption rates were evaluated. In addition, the respiratory control (RC) rate was calculated as the state 3/state 4 ratio of the respiratory rate. For the I/R protocols, ischemia was induced for 30 min followed by 60 min of reperfusion. No significant alterations were observed in the respiration in state 4, or resting respiration, between the tested groups. However, in the HT + I/R group, there was a significant increase in active oxygen consumption–state 3–as compared to the I/R group. It should be noted that the I/R group suffered a 54% decrease in O_2_ consumption values in state 3 as compared to the control (*p* < 0.01). On the contrary, the HT + I/R group exhibited state 3 O_2_ consumption levels comparable to those obtained for the control and HT groups. Data are presented as mean ± standard error of the mean (SEM). Statistical significance is indicated as follows: * *p* < 0.01 indicates a significant effect across groups in the overall analysis; # *p* < 0.001 vs. control (n _control_ = 3; n _I/R group_ = 3; n _HT_ = 2; n _HT + I/R group_ = 9).

In order to evaluate mitochondrial function, oxygen consumption was assessed in different metabolic states. As is shown in Figure 3, no significant alterations were observed in the respiration in state 4, or resting respiration, between the tested groups. However, in the HT + I/R group, there was a significant increase in active oxygen consumption—state 3—as compared to the I/R group. It should be noted that the I/R group suffered a 54% decrease in O_2_ consumption values in state 3 as compared to the control (243 ± 48 vs. 111 ± 31, *p* < 0.01). On the contrary, the HT + I/R group exhibited state 3 O_2_ consumption levels comparable to those obtained for the control and HT groups. This observation is consistent with the results of respiratory control (RC), where an increase was obtained for the HT + I/R and HT groups compared to the I/R group. These results suggest that acute treatment with HT protects the heart against mitochondrial uncoupling associated with I/R damage.

#### 3.2.2. Mitochondrial Hydrogen Peroxide Production

**Figure 4:**
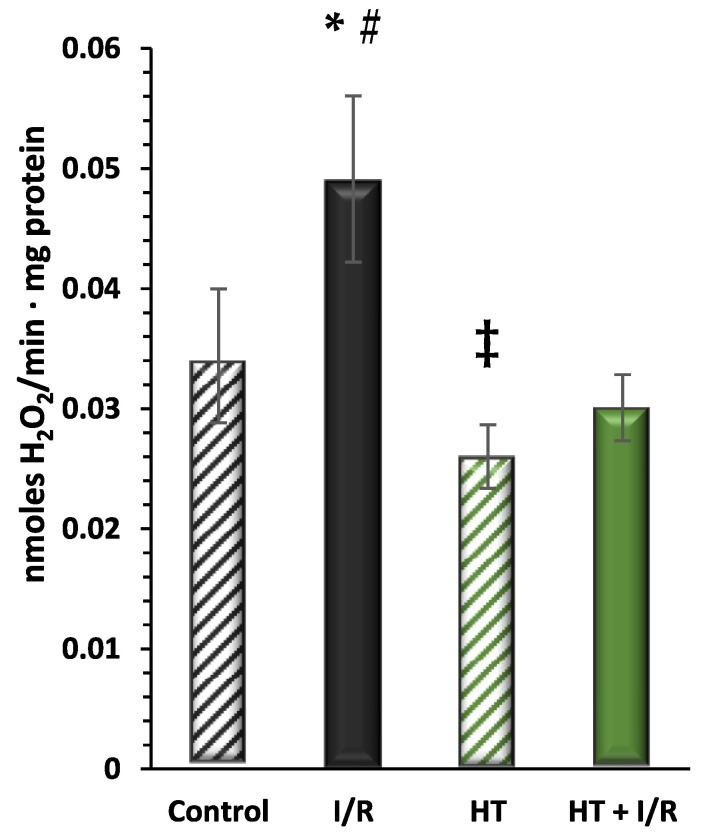
Hydrogen peroxide (H_2_O_2_) production measured using the Amplex Red assay. The figure shows H_2_O_2_ production rates in the experimental groups: control (untreated mice), I/R (mice subjected to I/R), HT (mice treated with HT at the specified dose), and HT + I/R (mice treated with HT at the specified dose and subjected to I/R). For the I/R protocols, ischemia was induced for 30 min followed by 60 min of reperfusion. The I/R group exhibited the highest H_2_O_2_ production as compared to controls. Notably, the HT + I/R group showed lower production levels as compared to the control group. Furthermore, the HT group displayed the lowest H_2_O_2_ production. Data are presented as mean ± standard error of the mean (SEM). Statistical significance is indicated as follows: * *p* < 0.05 indicates a significant effect across groups in the overall analysis; # *p* < 0.001 vs. control; ^‡^ *p* < 0.001 vs. I/R (n _control_ = 3; n _I/R group_ = 5; n _HT_ = 9; n _HT + I/R group_ = 11).

Next, the redox balance of the mitochondrial preparations was evaluated. Figure 4 shows the results obtained for the production of H_2_O_2_, as a molecule related to the incomplete reduction of O_2_ in the electron transport chain, of the investigated groups. These data are consistent with previous analyses, wherein the I/R group exhibited the highest H_2_O_2_ production as compared to controls (0.049 ± 0.007 vs. 0.034 ± 0.006). Notably, the HT + I/R group showed lower production levels as compared to the control group (HT + I/R 0.030 ± 0.003 vs. control 0.034 ± 0.006). Furthermore, the HT group displayed the lowest H_2_O_2_ production (0.026 ± 0.002), suggesting the antioxidant effect of the treatment, even in the absence of the insult by I/R.

#### 3.2.3. Mitochondrial Inner Membrane Potential

**Figure 5:**
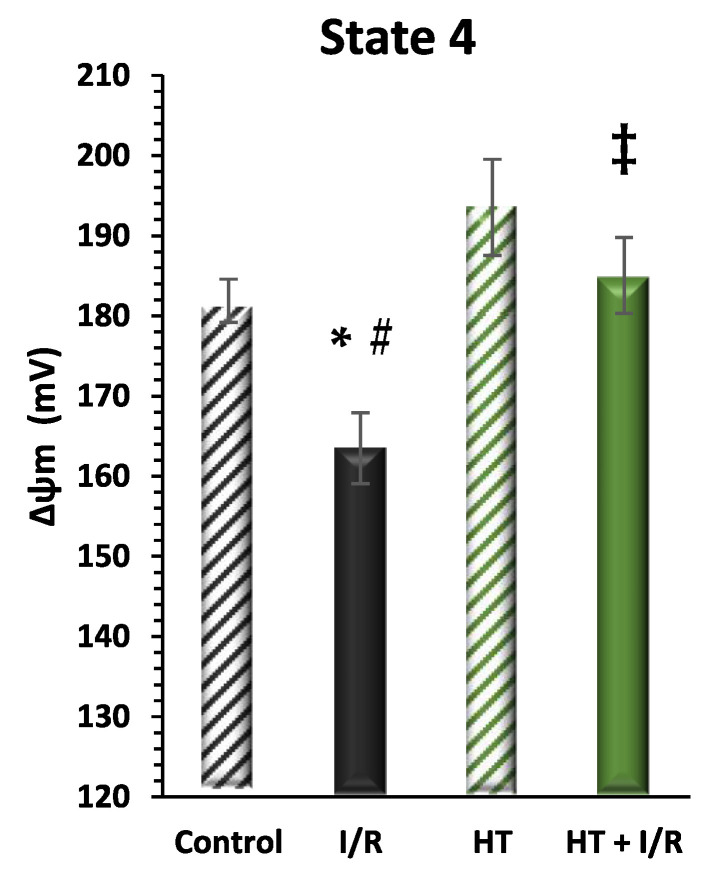
Mitochondrial membrane potential (ΔΨ_m_) in state 4 respiration measured using the rhodamine assay. The figure shows ΔΨ_m_ in four experimental groups: control (untreated mice), I/R (mice subjected to I/R), HT (mice treated with HT at the specified dose), and HT + I/R (mice treated with HT at the specified dose and subjected to I/R). For the I/R protocols, ischemia was induced for 30 min followed by 60 min of reperfusion. It should be noted that the HT + I/R group maintained ΔΨ levels comparable to those of the control group, in contrast to the decrease of about 10% observed in the I/R group. In accordance with the previous findings, the HT group once again showed significantly higher ΔΨ values (193 ± 5), thus indicating a protective effect of HT upon mitochondrial coupling in basal conditions. Data are presented as mean ± standard error of the mean (SEM). Statistical significance is indicated as follows: * *p* < 0.05 indicates a significant effect across groups in the overall analysis; ^#^ *p* < 0.001 vs. control; ^‡^ *p* < 0.001 vs. I/R (n _control_ = 3; n _I/R group_ = 5; n _HT_ = 4; n _HT + I/R group_ = 4).

Finally, mitochondrial function was evaluated considering mitochondrial inner membrane potential levels (ΔΨ) during mitochondrial respiration in state 4. Figure 5 shows a recurrence of statistically significant differences in the same direction as the data from the indirect assessment of ΔΨ through the evaluation of mitochondrial respiration in state 4 (Figure 3). It should be noted that the HT + I/R group maintained ΔΨ levels comparable to those of the control group (HT + I/R: 185 ± 5 vs. control: 182 ± 3), in contrast to the decrease of about 10% observed in the I/R group (163 ± 4). In accordance with the previous findings, the HT group once again showed significantly higher ΔΨ values (193 ± 5), thus indicating a protective effect of HT upon mitochondrial coupling in basal conditions.

#### 3.2.4. Mitochondrial Respiratory Complex Activity

**Figure 6:**
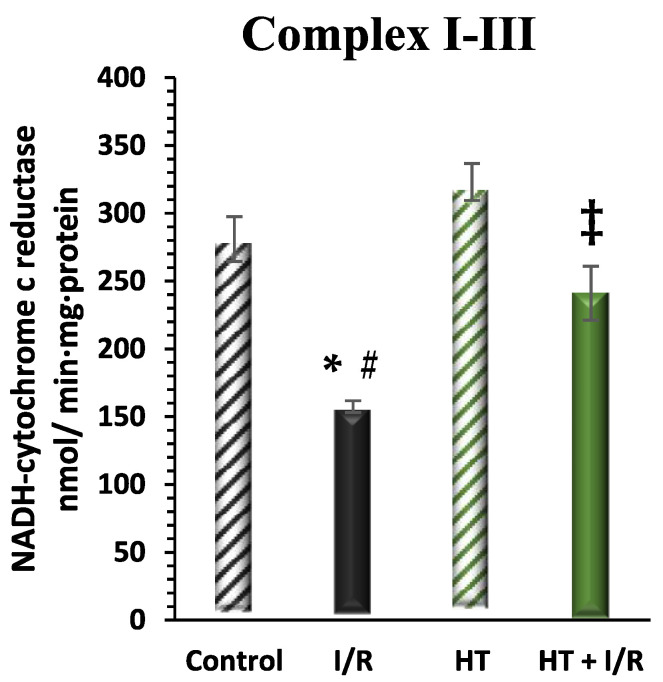
Activity of mitochondrial Complex I in the left ventricle of mice subjected to isolated heart I/R. The figure displays Complex I activity across four experimental groups: control (untreated mice), I/R (mice subjected to I/R), HT (mice treated with HT at the specified dose), and HT + I/R (mice treated with HT at the specified dose and subjected to I/R). For the I/R protocols, ischemia was induced for 30 min followed by 60 min of reperfusion. A notable increase in this activity was detected in the HT + I/R group compared to the I/R group, reaching levels similar to those of the control group. Treatment with HT administered 24 h prior to euthanasia resulted in a significant increase in Complex I activity in the group not subjected to the I/R protocol. Data are presented as mean ± standard error of the mean (SEM). Statistical significance is indicated as follows: * *p* < 0.05 indicates a significant effect across groups in the overall analysis; ^#^ *p* < 0.001 vs. control; ^‡^ *p* < 0.001 vs. I/R (n _control_ =3; n _I/R group_ =3; n _HT_= 5; n _HT + I/R group_ = 10).

The activity of mitochondrial respiratory Complex I was examined in the left ventricle (Figure 6). A notable increase in this activity was detected in the HT + I/R group compared to the I/R group (HT + I/R 241 ± 19 vs. I/R 157 ± 4), reaching levels similar to those of the control group (281 ± 16). Furthermore, once again, treatment with HT administered 24 h prior to euthanasia resulted in a significant increase in Complex I activity in the group not subjected to the I/R protocol (323 ± 14).

## 4. Discussion

It is now recognized that oxidative stress-induced mitochondrial damage plays a pivotal role in the pathogenesis of cardiovascular diseases [34], particularly in I/R injury [25,26]. Mitochondrial redox imbalance not only contributes to genetic disorders caused by inherited mitochondrial DNA mutations but is also involved in damage associated with I/R damage, as well as inflammation, aging, carcinogenesis, and neurodegenerative diseases. Given their central role in cellular energy metabolism, signaling, and programmed cell death, mitochondria are promising targets for antioxidant therapeutics [20,35,36].

The antioxidant and biological potential of polyphenols are rooted in their molecular structure, including the number and position of hydroxyl groups, availability of phenolic hydrogen, and ability to stabilize phenoxyl radicals through hydrogen donation or electron delocalization [37]. HT, a phenolic compound found in olive leaves, olives, and EVOO, features a hydroxyl group in the ortho position. In our study, we clearly observed that the administration of the phenolic compound HT at a single dose of 10 mg/kg via intraperitoneal injection to C57 mice, conducted 24 h prior to euthanasia for the subsequent I/R protocol, resulted in several beneficial effects. These included a reduction in infarct size and an improvement in mitochondrial function as evidenced by an increase in mitochondrial membrane potential, an elevation in the oxygen consumption rate, and a decrease in mitochondrial H_2_O_2_ production compared to untreated animals. These findings strongly suggest that HT could provide protection against AMI.

Pharmacological conditioning aims to protect the heart from myocardial I/R injury. Despite extensive research in this field, a significant gap persists between experimental findings and clinical practice. For instance, although volatile anesthetic-induced cardioprotection has shown promising results in experimental models, robust clinical evidence supporting its protective effects remains limited [38,39]. One of the primary challenges in translating preclinical discoveries into clinical applications is the failure to account for patient-related factors, such as risk factors, comorbidities, concomitant medications, and perioperative treatments. These variables—including aging, diabetes, hyperglycemia, metabolic syndrome, hypertension, statins, β-blockers, metformin, GLP-1 agonists, SGLT2 inhibitors, heparin, aspirin, and platelet inhibitors—can all interfere with cardioprotective interventions [40,41,42].

Interestingly, recent studies have suggested that comorbidities such as hypercholesterolemia, prediabetes, and metabolic syndrome may impair cardioprotective interventions, including insulin treatment and ischemic pre- and postconditioning [43]. This highlights the need for alternative strategies capable of restoring these protective pathways.

In this study, the results revealed a notable reduction in infarct size, with a 57% decrease observed in the HT-treated group compared to untreated mice (Figure 2). Clinically, limiting the infarct size is critical for preventing concomitant left ventricular dysfunction, which is a major determinant of long-term morbidity and mortality in patients following AMI and mortality in patients. HT has the potential to reduce the burden of heart failure, which frequently develops post-AMI. Moreover, limiting the myocardial infarct size could prevent adverse cardiac remodeling, reduce the incidence of heart failure, and improve patient outcomes [44,45,46].

These findings are corroborated by the research of Miao et al. [47], who also observed a reduction in infarct size with low doses of HT. Specifically, Miao et al. [47] perfused isolated hearts with 10 μM HT for 10 min, followed by K-H solution for 5 min before inducing ischemia, suggesting a dose-dependent effect of HT at lower concentrations (10–100 μM), consistent with a previous study by Pan et al. [23]. Additionally, Pei et al. [25] conducted in vivo experiments on Sprague–Dawley rats subjected to 30 min of cardiac ischemia followed by 3 h of reperfusion. Their study evaluated HT doses of 1, 5, and 20 mg/kg, identifying 20 mg/kg as the most effective in reducing infarct size when administered intraperitoneally just 5 min before ischemia. Based on these findings, we selected a dose of 10 mg/kg in our study, adjusting for our pre-treatment protocol of 24 h. This extended pre-treatment period was designed to allow HT sufficient time to exert its systemic antioxidant and mitochondrial protective effects, potentially compensating for the lower dose while ensuring efficacy and translational feasibility. Unlike these previous models, our approach employed a single dose of HT administered 24 h before the I/R protocol, allowing us to evaluate its preventive potential and providing a more translational perspective on its application in clinical settings. The promising results obtained with single-dose administration highlight the potential of HT as a therapeutic agent and suggest that continuous administration or long-term regimens could further enhance its efficacy. However, additional studies are required to determine the optimal dosing strategy for sustained cardioprotective effects.

We also focused our study on the bioenergetic state of the heart, observing that the mitochondria in the HT-treated mouse groups exhibited a significant increase in function evidenced both in state 3 respiration and in respiratory control (RC) values. State 4 respiration represents a model of resting metabolism, while state 3 reflects active metabolism. Remarkably, the group treated with HT without the I/R protocol demonstrated the highest values in both state 3 and RC (Figure 3). This outcome is logical as it indicates a greater activity compared to the control group itself, thereby demonstrating the direct effect of HT on heart energy metabolism. Conversely, the group treated with HT + I/R displayed state 3 levels comparable to those of the control group. This finding underscores the protective effect of HT against reperfusion injury, as it retains mitochondrial activity even under conditions of I/R. It has been reported that phenolic compounds, such as resveratrol and derivatives of caffeoylquinic acid (CQA), can enhance mitochondrial activity. For instance, a mixture of resveratrol with leucine has been shown to increase mitochondrial activity. Studies conducted in adipocytes have demonstrated that the resveratrol–leucine mixture enhances the oxygen consumption rate following palmitate incubation, indicating an induction of fatty acid oxidation [48]. Additionally, derivatives of CQA obtained from purple sweet potato have been found to augment mitochondrial maximal respiration and increase fatty acid oxidation in mouse primary hepatocytes [49].

Furthermore, our study demonstrated that mice treated with HT exhibited a reduced rate of mitochondrial H_2_O_2_ production. This difference was evident even between the control group and the HT group, where the HT group displayed the lowest rate of H_2_O_2_ production (Figure 4). These findings highlight the cardioprotective effect of HT against ischemic damage, as well as the improved redox balance in animals treated with HT. While our study focused on the acute effects of a single HT dose, future research should address whether these antioxidative properties are sustained over time with repeated administration, which would have important implications for long-term cardiovascular protection. Regarding the molecular mechanisms involved in cardioprotective processes, Namik et al. [50] demonstrated that HT attenuates xanthine/xanthine oxidase (X/XO)-induced toxicity in H9c2 cardiomyocytes by the regulation of oxidative stress and stress-sensitive signaling pathways. These authors showed that western blotting experiments revealed X/XO-induced increases in the phosphorylation of downstream substrates of p38, MAPK-activated protein kinase 2, p44/42-MAPK (Erk1/2), and cleaved caspase-3. Taken together with our data, we hypothesize that HT could protect against myocardial infarction by potentially diminishing intracellular ROS levels and modulating stress-sensitive protein kinase cascades and transcription factors in our ischemia/reperfusion model. These results are consistent with the findings of other studies investigating the effects of phenolic compounds on H_2_O_2_ production rates. For instance, Calabriso et al. [20] observed that HT exerts its protective effects by preserving mitochondrial function, reducing oxidative stress, and enhancing endothelial cell function. Additionally, other studies have shown similar effects of phenolic compounds on H_2_O_2_ production. For example, Lagoa et al. [51] found that different flavonoids have varying effects on H_2_O_2_ production by brain and heart mitochondria, with quercetin and kaempferol exhibiting the most potent inhibitory effects on H_2_O_2_ production with IC50 values in the low micromolar range. Similarly, Xing et al. [52] demonstrated that salidroside treatment mitigates H_2_O_2_-induced endothelial dysfunction by reducing oxidative stress and enhancing endothelial cell function.

The mitochondrial membrane potential (ΔΨm) serves as a critical indicator of mitochondrial function, with a decrease typically indicating mitochondrial damage. In our study, we assessed the alterations in ΔΨm in mouse isolated mitochondria and found that HT treatment led to an increase in ΔΨm. Remarkably, even under an I/R protocol, the ΔΨm levels were comparable to those of the control group, thereby confirming the beneficial effects of HT on mitochondrial function (Figure 5). These findings are in accordance with the results reported by Dong et al. [53], where HT was shown to enhance mitochondrial function by activating mitophagy in seabass.

Mitochondrial Complex I is of paramount importance in cellular function and human health. This intricate assembly plays a critical role in the electron transport chain, facilitating the generation of ATP, which serves as the primary energy source for cells. Our study shows that treatment with HT enhances the activity of Complex I, thereby enabling ATP production. Moreover, our findings consistently demonstrate that the HT + I/R group attains activity levels comparable to those of the control group, while the HT-treated group exhibits the highest Complex I activity (Figure 6). This observation aligns with the research conducted by Hao et al. [54], who also reported an increase in Complex I activity following HT administration.

**Figure 7:**
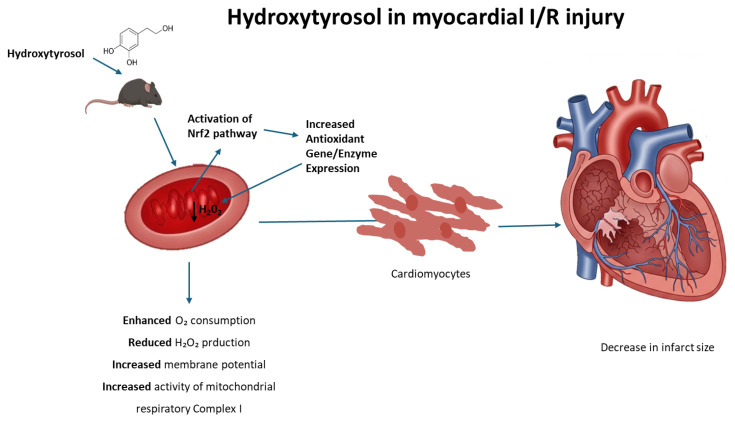
Proposed mechanism of hydroxytyrosol (HT) cardioprotection against ischemia/reperfusion (I/R) injury. Hydroxytyrosol (HT) acts within the cardiomyocyte to mitigate I/R damage. Upon entering the cell, HT activates the Nrf2 pathway, leading to its nuclear translocation and subsequent increased expression of antioxidant genes (ARE). The enhanced antioxidant defense system, along with HT’s direct effects, results in reduced oxidative stress (decreased H_2_O_2_). This action, coupled with improved mitochondrial function (evidenced by increased respiration, enhanced mitochondrial potential, and improved mitochondrial complex activity), collectively leads to cardioprotection and a reduction in myocardial infarct size.

During heart I/R injury, the overproduction of reactive oxygen species is stimulated. In the first phase, the absence of oxygen arrests oxidative phosphorylation, which establishes mitochondrial membrane depolarization, ATP depletion, and the inhibition of contractility in cardiomyocytes. In this context, cellular metabolism shifts to anaerobic glycolysis, resulting in the accumulation of lactate, which leads, in turn, to intracellular calcium overload. During reperfusion, the electron transport chain is reactivated, generating excessive ROS, which is a key factor in the loss of mitochondrial functionality and cellular viability. In this context, mitochondrially targeted antioxidants, such as MitoQ, have been tested to protect mitochondria and the heart against the deleterious effects of reperfusion [55]. Nevertheless, the protective effects on I/R models observed with molecules such as HT and other phytochemicals have been repeatedly attributed to their ability to activate the transcription factor nuclear factor erythroid 2-related factor 2 (Nrf2), which is believed to control the basal and inducible expression of over 1000 genes involved in antioxidant defense [56]. Interestingly, Nrf2 activates the expression of genes containing antioxidant response element (ARE) sequences in their promoters, including those coding for enzymes involved in the generation of NADPH, an essential cofactor for antioxidant reactions [57]. As an example, resveratrol, a plant polyphenol found in some plants including grapes and berries, exhibits cardioprotective effects upon I/R that include the upregulation of endogenous enzymatic and non-enzymatic antioxidants attributable to Nrf2 activation [58]. Likewise, rosemary derived carnosic acid was shown to improve heart dysfunction by activating antioxidant defense systems through the Nrf2 pathway in isoproterenol-induced myocardial injury [59]. More recently, Liu et al. [60] reported that mitochondria-targeted triphenylphosphonium (TPP+)-HT prevents endothelial dysfunction by enhancing mitochondrial function and redox balance by promoting Nrf2 nuclear translocation [60]. Taking this evidence into account, the molecular mechanism that explains the effects of HT in mitochondrial function could be potentially attributed to the ability of this compound to act as a pharmacological activator of Nrf2, thus preventing the deleterious effects of the redox imbalance that takes place in the mitochondrial environment during heart I/R; consequently, we can deduce that HT not only emerges as a remarkable candidate for safeguarding the heart against ischemic injury but also augments mitochondrial function and preserves redox balance in control hearts. The observed cardioprotective effects after a single-dose administration reinforce the potential of HT as a promising therapeutic compound. While further studies are necessary to evaluate the long-term impact and optimal dosing strategy, our findings provide a strong foundation for future research aimed at its clinical translation. This underlines the broader therapeutic potential of HT beyond cardiac protection, encompassing the preservation of overall cellular health and function (Figure 7).

Although this study provides promising evidence regarding the cardioprotective effects of HT in a murine ischemia/reperfusion (I/R) model, it also exhibits some limitations. The animal model, while useful for investigating the mechanisms of action, does not fully replicate the complexity of human cardiovascular disease, requiring validation in larger models and clinical trials. Additionally, the single-dose regimen tested in this study should be considered as an initial step, as it is unclear whether the observed effects would persist chronically or if repeated treatments would be necessary. Furthermore, while this study focused on mitochondrial function and oxidative stress, further exploration of other mechanisms involved in cardiovascular protection would be beneficial. Despite these limitations, the results obtained are highly promising and justify further research to optimize dosing regimens and explore the therapeutic potential of HT for treating cardiovascular diseases.

## 5. Conclusions

In conclusion, our study sheds light on the promising therapeutic potential of HT in protecting the heart against AMI consequences. Through a comprehensive analysis of various parameters, we observed significant benefits associated with HT treatment in a murine model of I/R injury. Our findings indicate that HT treatment resulted in a reduction in myocardial infarct size, increased mitochondrial membrane potential, elevated oxygen consumption rate, and decreased production of hydrogen peroxide compared to untreated animals. These effects suggest that HT may confer protection against acute ischemia/reperfusion injury by enhancing mitochondrial function and reducing oxidative stress. Additionally, HT treatment demonstrated a notable increase in mitochondrial function, particularly in metabolic state 3 and respiratory control, highlighting its role in preserving mitochondria even under conditions of I/R injury. Furthermore, our findings suggest that HT treatment leads to a reduction in mitochondrial hydrogen peroxide production, indicative of improved redox balance and decreased oxidative stress. These results are in line with previous studies investigating the effects of phenolic compounds on mitochondrial function and oxidative stress. Unlike previous studies, which primarily administered HT minutes before ischemia or directly to the heart via perfusion, our model involves the systemic intraperitoneal administration of HT at a dose of 10 mg/kg, delivered 24 h prior to the I/R protocol. This approach allows us to evaluate the preventive potential of HT in a whole-organism context, offering a more clinically relevant perspective on its therapeutic application.

## Data Availability

The data presented in this study are available in the article.
